# Bidirectional interaction between intestinal microbiome and cancer: opportunities for therapeutic interventions

**DOI:** 10.1186/s40364-020-00211-6

**Published:** 2020-08-12

**Authors:** Dibyendu Dutta, Seah H. Lim

**Affiliations:** 1 Division of Hematology and Oncology, SUNY Downstate Health Sciences University, 450 Clarkson Avenue, Room B5-495, Brooklyn, New York 11203 USA; 2 Division of Hematology and Oncology, Department of Medicine, New York Medical College, Valhalla, New York USA

**Keywords:** Intestinal dysbiosis, Cancer development, Cancer therapy, Microbial therapy

## Abstract

Gut microbiota composition influences the balance between human health and disease. Increasing evidence suggests the involvement of microbial factors in regulating cancer development, progression, and therapeutic response. Distinct microbial species have been implicated in modulating gut environment and architecture that affects cancer therapy outcomes. While some microbial species offer enhanced cancer therapy response, others diminish cancer treatment efficacy. In addition, use of antibiotics, often to minimize infection risks in cancer, causes intestinal dysbiosis and proves detrimental. In this review we discuss the role of gut microbiota in cancer development and therapy. We also provide insights into future strategies to manipulate the microbiome and gut epithelial barrier to augment therapeutic responses while minimizing toxicity or infection risks.

## Background

Human intestinal microbiota is essential for microbial homeostasis, regulation of metabolism, and immune tolerance. Intestinal dysbiosis occurs when there are altered ratios of healthy microbial flora along with changes in their diversity and density. Such changes may lower mucus layer thickness, reduce antimicrobial defense, and disrupt the epithelial tight-junction barriers to allow increased translocation of intestinal bacteria and bacterial products into the systemic circulation and trigger inflammation and immune responses. Circulating bacterial products such as endotoxin, genotoxin and trimethylamine oxide have been implicated in many human disorders, including metabolic syndrome, cardiovascular complications (atherosclerosis and thrombosis), and various neoplastic conditions. Intestinal dysbiosis may also affect adaptive immunity by modulating the functions of T lymphocytes and promoting tumor immune escape.

While increased translocation of intestinal luminal content is associated with carcinogenesis and poor therapeutic response, the cause-effect relationship is often bidirectional. In this review we will discuss the role of gut microbes in modulating tumor immunity, intestinal permeability and cancer development. Next, we will highlight the effects of intestinal dysbiosis and increased permeability in cancer therapy. Finally, we will explore the options to improve gut health to enhance the efficacy of cancer therapy.

## Intestinal immunity and permeability

**Fig. 1:**
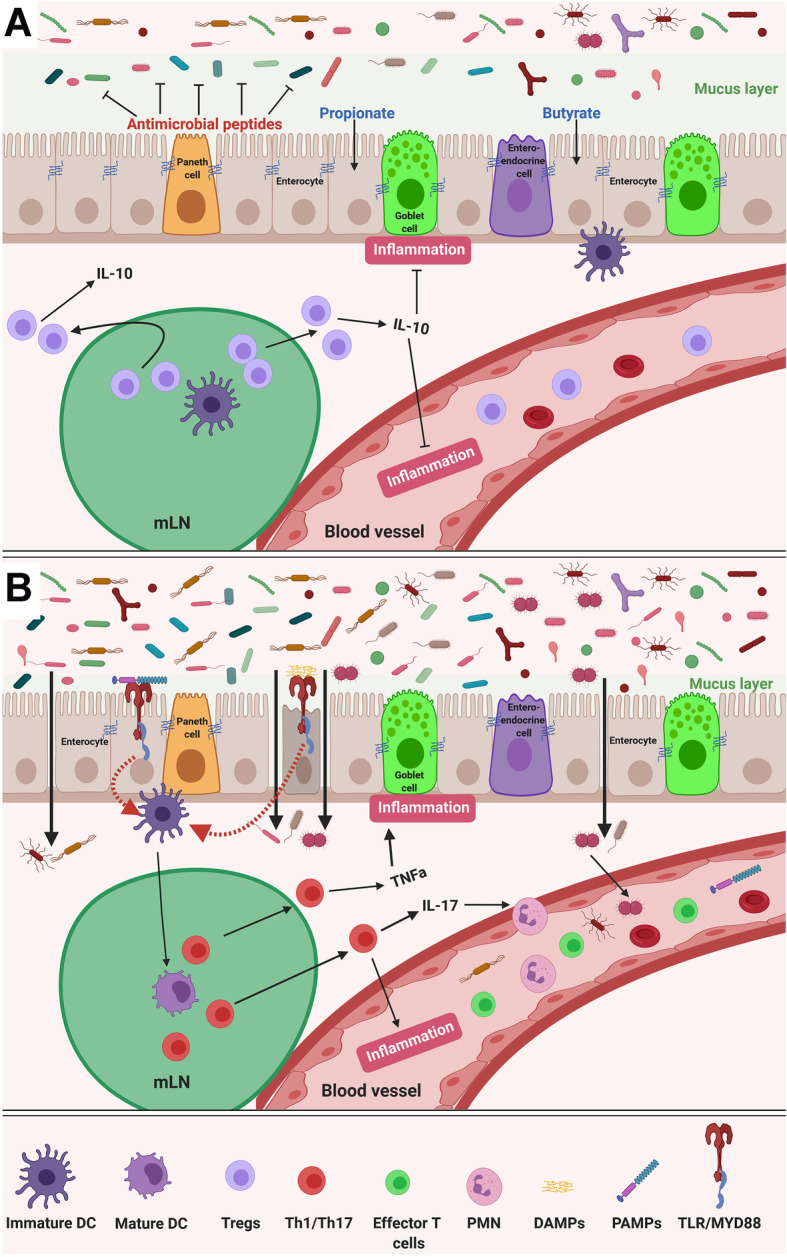
Interplay between different factors involved in gut immunity and permeability. **a** The intestinal epithelial cells containing Paneth cells, goblet cells, enterocytes and enteroendocrine cells coordinate with intra-epithelial lymphocytes to generate a functional immune response. Paneth cells secrete antimicrobial peptides and goblet cells produce mucus to cover the epithelial layer. This mucus layer prevents adhesion of microbes to the epithelial cells. Lamina propria situated under the mucus layer contains Peyer’s patches and immune cells including antigen presenting cells (APCs) like dendritic cells (DCs), T cells and B cells. Pattern recognition receptors (PRRs) such as toll-like receptors (TLRs) on epithelial cells interact with microbe-derived pathogen-associated-molecular patterns (PAMPs) such as lipopolysaccharide (LPS) to activate MYD88-dependent signaling. DCs travel to mesenteric lymph nodes (mLN) and promote the differentiation of naïve T cells to regulatory T (T_reg_) cells that migrate to other sites. T_reg_ cells secrete IL-10 to elicit an anti-inflammatory response. **b** Dysbiosis decreases mucus layer thickness and short-chain fatty acid (SCFAs) production. This affects the secretion of antimicrobial peptides and allows microbes to come in close proximity to the epithelial cells. Reduction in SCFAs influences gut barrier dysfunction. As a result, the gut luminal content also translocated and spreaded through the systemic circulation to trigger local and systemic immune responses. In addition to PAMPs, DAMPs released from damaged intestinal epithelium interact with PRRs to facilitate expression of macrophages and maturation of DCs. Mature DCs promote the differentiation of naïve T cells to effector T cells such as T helper cells (Th1, Th2, Th17). Th1 release TNFα and IFNγ, and Th17 secrete IL-17 to recruit polymorphonuclear neutrophils (PMNs). These cytokines create a pro-inflammatory condition

The intestinal architecture and microbiota regulate innate and adaptive immunity. Disruption of the architecture and/or microbiota affects these functions. The relationships between the different players in the intestinal microenvironment is summarized in Fig. 1.

The composition of microbes in the gut dictates mucus layer thickness and production of anti-microbial signals. In germ-free mice, mucus layer and effector T cells are absent [1, 2]. Microbes secrete short-chain fatty acids (SCFAs) such as propionate and butyrate that prevent microbial binding to the epithelial cells and help maintain barrier function and immune homeostasis. Butyrate promotes tight-junction formation [3, 4], and activates peroxisome proliferator-activated receptor gamma (PPAR-γ) to enhance epithelial oxygen consumption, resulting in reduced emanation of oxygen from the mucosal surface. It helps in maintaining an anaerobic condition in the gut lumen needed for colonization of obligate anaerobes [5]. This intestinal microenvironment determines the composition of resident bacterial species. For example, only *Clostridium*, *Lactobacillus* and *Enterococcus* are enriched on the epithelial surface and in the mucus layer, whereas *Bacteroides*, *Bifidobacterium*, *Streptococcus*, Enterobacteriaceae, *Enterococcus*, *Clostridium* and *Lactobacillus* are all predominant in the intestinal lumen [6].

Dysbiosis increases inflammatory signals that shift the metabolism of enterocytes. Epithelial hypoxia is eliminated and increased oxygenation results in the release of more oxygen from the mucosal surface. Since only facultative anaerobes can respire oxygen, dysbiosis-induced shift in epithelial oxygenation alters gut microbial community from obligate to facultative anaerobes [5]. Intestinal pathogens, such as *Proteobacteria*, produce genotoxins like colibactin and cytolethal distending toxin (CDT) to induce inflammation and host deoxyribonucleic acid (DNA) damage that initiates tumor formation [7]. Dysbiosis also decreases mucus layer thickness, reduces SCFA production, and damages mucosal barrier, allowing pathogen-associated-molecular patterns (PAMPs) to interact with pattern recognition receptors (PRRs) and activate Toll-like receptor (TLR) 2/4-Myeloid differentiation primary response protein 88 (MYD88) signaling pathways. In addition, changes in microbial composition and density triggers epithelial release of damage-associated molecular patterns (DAMPs), such as extracellular adenosine triphosphate (ATP), cytoplasmic calreticulin, high mobility group box 1 (HMGB1) proteins, endogenous nucleic acids, and intracellular proteins to interact with PRRs. PRR engagement triggers a pro-inflammatory condition that causes tissue damage and local inflammation. Microbiota-driven TLR immune signaling has been implicated in cancer formation and modification of treatment efficacy [8–11]. For example, CpG oligodeoxynucleotides that mimic bacterial DNA acts as a PAMP to trigger a TLR9-dependent TLR4 activation and tumor necrosis factor (TNF)-α production by tumor-infiltrating myeloid-derived cells [12]. Mice bearing EL4 lymphoma, MC38 colon carcinoma and B16 melanoma when treated with CpG oligodeoxynucleotides show reduced tumor growth and enhanced survival rate. The beneficial effects of CpG oligodeoxynucleotides were positively associated with the abundance of *Alistipes shaii* in the gut [12].

## Effects of intestinal microbiota on cancer development

Intestinal microbes can influence local and distant carcinogenesis through infection and microbial products, or by modulating tumor immunosurveillance. This is accomplished via altering the balance between the rate of cell proliferation and apoptosis, triggering chronic inflammation and/or immunosuppression, or changing the metabolism of the products produced by host and microbes. In this section, we will discuss how intestinal dysbiosis-related permeability may contribute to tumorigenesis in different organs.

### Colorectal cancer

*Fusobacterium nucleatum,* a Gram-negative mucosa-adherent anaerobic bacteria, has been implicated in the initiation and progression of colorectal cancer (CRC) [13, 14]. FadA, an adhesion molecule on *F. nucleatum*, binds to host E-cadherin to enter epithelial cells [13]. This activates the WNT/β-catenin pathway, leading to an increased secretion of inflammatory cytokines including IL-6, IL-8 and TNF-α, and upregulation of Nuclear Factor kappa light chain enhancer of activated B cells (NF-κB) that facilitates CRC development. In addition, it attracts myeloid-derived suppressor cells and the autotransporter protein Fap2 interacts with the human inhibitory receptor, T cell immunoreceptor with Ig and ITIM domains (TIGIT), to create a tumor immunosuppressive microenvironment. *F. nucleatum* may also induce chemoresistance by modulating the TLR4-MYD88 signaling pathway following 5-Fluoruracil treatment [11].

In CRC patients, an increased *abundance of F. nucleatum along with Clostridium difficile* and species of *Streptococcus*, *Campylobacter* and *Leptotrichia* has been demonstrated in tumor tissue and fecal materials [15–17]*. F. nucleatum-mediated colorectal carcinogenicity occurs downstream of* APC. Introduction of *F. nucleatum* resulted in rapid onset of colonic tumors in mice deficient in one copy of Adenoma Polyposis coli (*APC)* (*Apc*^*Min/+*^) gene [14]. Both intestinal dysbiosis and loss of APC disrupt epithelial tight-junctions and mucus layer [18, 19] and allow increased infiltration of *F. nucleatum* and other non-residential microbes to drive CRC development. The role of defective gut barrier in CRC has been confirmed in mucin 2-knockout (*Muc2*^−/−^) mice in which the lack of gastrointestinal mucin resulted in spontaneous CRC development [20]. Therefore, dysbiosis-induced gut permeability may play an important role in tissue enrichment of *F. nucleatum* and increased risks for CRC.

### Hepatobiliary cancer

The liver is chronically exposed to intestinal microbiota and its products via the portal vein. Intestinal dysbiosis and increased permeability enhance translocation of gut microbiota to trigger inflammation and chronic liver disease that predisposes patients to the development of hepatocellular cancer. Alteration in bile acid metabolism due to changes in *Clostridium* spp. suppress anticancer immunity [21]. In mice, eradication of Gram-positive bacteria by oral vancomycin inhibits secondary bile acid conversion, resulting in the upregulation of chemokine (C-X-C motif) ligand (CXCL)16 in liver sinusoidal endothelial cells. CXCR16 recruits natural killer T (NKT) cells in the tumor microenvironment and kill tumor cells in a CD1d-dependent manner. In addition, gut microbiota-derived lipopolysaccharides (LPS) promote tumor progression in liver cancer by activating the TLR4 signaling [8]. In a study involving 60 cholangiocarcinoma patients, bile duct tissues had distinct dominance of *Dietziaceae*, *Pseudomonadaceae* and *Oxalobacteraceae* members [22].

### Pancreatic cancer

Gut microbiota influences the development of pancreatic cancer through activating TLR4 signaling [23]. The stroma in pancreatic tumor harbors an abundance of microbiota, especially *Bifidobacterium pseudolongum*, compared to normal pancreas [24]. This helps in creating an immunosuppressive environment by differentially activating distinct TLRs in monocytes. Pancreatic adenocarcinoma *has an enrichment of Proteobacteria, Synergistetes,* and *Euryarchaeota* [24]. Longer survival is observed in patients with a more diverse intratumor microbial composition, primarily of *Sachharopolyspora*, *Pseudoxanthomonas*, *Streptomyces*, and *Bacillus clausii* [25]. Tumoral colonization with *Mycoplasma hyorhinis* and *Gammaproteobacteria* is associated with gemcitabine resistance [26]. Antibiotics diminish myeloid-derived suppressor cells and increase antitumor M1 macrophages to promote Th1 differentiation of CD4^+^ T cells and CD8^+^ T cell activation in the tumor [24]. Co-treatment of gemcitabine with ciprofloxacin abrogated *Gammaproteobacteria*-induced chemotherapy resistance [26]. The efficacy of immune checkpoint inhibitors (ICIs) therapy is also enhanced by antibiotics [24].

### Lung cancer

While local microbiota is important [27], there are reports that gut microbiome may also contribute to lung cancer development. Lung cancer patients demonstrated an abundance in intestinal *Enterococcus* and depletion in *Bifidobacterium* and *Actinobacteria* [28]. They are also enriched with *Veillonella*, *Bacteroides*, and *Fusobacterium*, depleted of *Dialister, Enterobacter, Escherichia-Shigella, Fecalibacterium*, and *Kluyvera* [29]. In non-small cell lung cancer (NSCLC) patients, butyrate producers such as *Faecalibacterium prausnitzii*, *Clostridium leptum*, *Clostridial cluster I*, *Ruminococcus* spp., *Clostridial cluster XIVa*, and *Roseburia* spp. were significantly reduced [30]. Since butyrate is essential for preserving mucosal homeostasis, reduction of intestinal butyrate producers may imply a compromised intestinal barrier in these patients.

### Hematologic malignancies

Dysbiosis-induced intestinal permeability affects mucosa-associated lymphoid tissue (MALT) and plays a significant role in hematologic malignancies. Composition of intestinal microbiota is responsible for maintaining the pool of bone marrow myeloid cells [31]. Pre-leukemic myeloproliferation is driven by microbial signals in ten-eleven translocation-2 (*Tet2*)-deficient mice [32, 33]. These mice show increased infiltration of inflammatory cells, disrupted mucosal barrier and increased translocation of bacteria [32, 34]. It was suggested that dysfunction of small intestinal barrier and leakage of microbes can occur due to *Tet2* mutation in hematopoietic compartment [32]. Occurrence of *Tet2* mutation, intestinal dysbiosis and leaky gut is common in leukemia and lymphoma.

Acute myeloid leukemia (AML) and acute lymphoblastic leukemia (ALL) patients have a compromised intestinal barrier [35–37]. Fecal microbiota in ALL patients showed lower microbial diversity [38]. They were enriched in *Enterococcaceae*, *Porphyromonadaceae*, and *Bacteroidetes* (mainly *B. fragilis*), and depleted in *Blautia*, *Erysipelotrichiales*, *Lachnospiraceae* and *Clostridiales* members [39, 40]. Abundance of *Staphylococcaceae* and *Streptococcaceae* have also been reported in pediatric ALL and adult AML [41, 42].

*Helicobacter pylori* is associated to MALT lymphoma [43], and *Chamydophila psittaci* to ocular MALT lymphoma [44]. While *Borrelia burgdorferi* was linked to cutaneous B-cell non-Hodgkin lymphoma [45], two studies did not find significant risk of *Borrelia burgdorferi* in the development of non-Hodgkin lymphoma [46, 47]. Abundance of *Proteobacteria* is a predictor for neutropenic fever, and enrichments of *Enterococcaceae* and *Streptococcaceae* are strong predictors of infectious complications in ALL [42]. Similarly, higher gut microbiota diversity in multiple myeloma is associated with reduced risk for disease relapse [48]. ALL patients with infectious complications have an abundance of *Brevundimonas diminuta* and *Agrobacterium tumefaciens*, whereas *Faecalibacterium prausnitzii* (producer of SCFAs) is completely absent [49]. Similar findings have been reported in non-Hodgkin lymphoma with infectious complications [50].

## Effects of intestinal microbiota on cancer therapy

The efficacy of cancer treatment is, in parts, dependent on normal immune function. Since gut microbiota plays a crucial role in modulating immune response, it is not surprising that dysbiosis affects treatment outcomes. Prophylactic antibiotics are commonly used for cancer patients undergoing chemotherapy and allogeneic hematopoietic stem cell transplantation (allo-HSCT) to reduce the risk of neutropenia-associated infection. However, antibiotic use causes intestinal dysbiosis that results in negative outcomes, including poor treatment response and toxicity, and the development of *Clostridium difficile infection (CDI).* In addition to antibiotics, opioid analgesics for cancer pain management may also trigger dysbiosis. Opioid analgesics impair intestinal motility and promote bacterial overgrowth resulting in dysbiosis and gut permeability [51].

Intestinal dysbiosis induces mucosal injury and triggers the release of DAMPs. DAMPs have a dual and bidirectional effect on cancer. Although DAMPs exert immunosurveillance and immune-mediated cell death to eliminate tumor cells and protect against cancer development, chronic inflammation induced by DAMPs may promote tumor initiation. DAMPs released by apoptotic cells from cancer therapy may also induce chemoresistance and promote metastasis. For example, TLR7/8 expressed on tumor cells may bind DAMPs (loxoribine for TLR7, and poly U for TLR8) and promote chemoresistance through the activation of NF-κB and the upregulation of BCL2 [52]. DAMPs may also activate TLR9 on human breast, prostate and lung cancer cells to trigger tumor invasion and metastasis [53, 54]. Given the clinical significance of dysbiosis-mediated mucosal injury and permeability in cancer, we will, in this section, discuss how the treatment outcome by various cancer therapy may be affected by intestinal microflora and permeability.

### Chemotherapy and radiation therapy

Intestinal microbial composition and mucosal barrier function influence chemotherapeutic outcome, and the effect is bidirectional. While dysbiosis can exacerbate chemotherapy drug toxicity and reduce its efficacy, chemotherapy can itself cause dysbiosis. Although, prevalence of certain intestinal microbes in the gastrointestinal tract offer beneficial effects, others contribute to chemoresistance and drug toxicity. This multiple-pathway effect is best covered by TIMER mechanisms [55] – *T*ranslocation of microbes; *I*mmunomodulation; *M*etabolism and *e*nzymatic effects on drugs; and *R*educed microbial diversity. These mechanistic effects alter chemotherapy efficacy and toxicity, and risks for infections. For example, translocation of microbes due to chemotherapy induced-dysbiosis and disruption of mucosal barrier can increase the risk of infection. However, certain chemotherapy drugs such as cyclophosphamide and doxorubicin damage intestinal barrier for the translocation of commensal bacteria into secondary lymph nodes to elicit anti-tumor immune response [55]. Vancomycin prophylaxis inhibits antitumor effects of cyclophosphamide in fibrosarcoma inoculated mice [56]. Irinotecan, used for CRC treatment, is transformed into its active form SN-38 by tissue carboxylesterase [55]. It is detoxified in the liver by host UDP-glucuronosyltransferases into inactive glucuronide (SN-38-G) and excreted into the gut via bile ducts. In the gut, bacterial β-glucuronidases reconverts SN-38-G into active SN-38, which causes severe intestinal toxicity and diarrhea [57]. Streptomycin inhibits irinotecan absorption and reduces epithelial carboxylesterase activity and diarrhea [58]. Ciprofloxacin inhibit β-glucuronidases [59] and low dose amoxapine (β-glucuronidases inhibitor) suppress irinotecan-associated diarrhea in rats [60]. Table 1 provides a selection of chemotherapeutic agents affecting and affected by intestinal microbial composition and permeability.

**Table 1:** Selection of chemotherapeutic agents and the bidirectional effects between the chemotherapy and intestinal microbiota

Chemotherapy Drug	Effects on Gut/ Changes in Microbiota	Toxicity/ Infection	Microbial Intervention
Cisplatin	Damages mucosal barrier by impairing DNA replication of rapidly proliferating epithelial cells [61] Facilitates translocation of gut bacteria Commensal gut bacteria influences genotoxicity by inducing reactive oxygen species (ROS) production and recruitment of pathogenic Th17 cells in the tumor microenvironment independently of immunity elicited by immunogenic cell death [12]	CDI [62] Ototoxicity [63]	Antibiotics against Gram-positive bacteria abrogate antitumor chemotoxicity, increase tumor size and decrease survival rate Cisplatin alone show better response compared to a combined treatment of cisplatin and antibiotics in mice with lung cancer [64]. The combination treatment increased tumor size and decreased survival rate *Lactobacillus acidophilus* restores antitumor efficacy following antibiotic treatment [64, 65] Restoration of gut microbiota and epithelial integrity by FMT [66] and treatment with D-methionine [67, 68] prevent infections and ototoxicity without affecting tumor chemotoxicity
Paclitaxel	Increases gut permeability, as indicated by 5-fold elevation in circulating LPS-binding protein and systemic inflammation [69] Reduces abundance of *Roseburia, Porphyromonadaceae* and *Akkermanisa Muciniphila* [69, 70]	Chemotherapy-induced peripheral neuropathic pain (CIPN) [70] CDI [71, 72]	FMT increases *A. Muciniphila* abundance and reduces CIPN [70]
5-Fluoruracil	Reduces *Clostridium* spp. and increases members of Proteobacteria, mainly *Enterobacteriaceae* [73] Damages mucosal barrier	Mucositis along the entire gastrointestinal tract [74] CDI [75, 76]	Oral butyrate supplementation improves gut barrier by reducing inflammation and mucositis [77] Antibiotics reduce mucositis and cytokine production but also diminish antitumor efficacy [78] and promote chemotherapy resistance [11]
Cyclophosphamide	Triggers disruption of gut barrier by altering bacterial composition Gram-positive bacteria such as *Enterococcus hirae*, *Lactobacillus johnsonii*, and *L. murinus* translocate from gut into mesenteric lymph nodes and spleen [56]. This enhances immune responses by the production of interferon gamma (IFN-γ) and activation of Th17 cells	CDI [75]	Antibiotics against Gram-positive bacteria reduce Th17 responses, and subsequent development of cyclophosphamide-resistance Re-establishment of *E. hirae* alone restores antitumor activity [79]. *E. hirae* decreases tumor-infiltrating T_regs_. *Barnesiella intestinihominis* accumulates in the colon and increases the number of intra-tumoral IFN-γ-producing γδT cells. *E. hirae* and *B. intestinihominis* synergistically stimulate local and systemic immunity to improve anticancer effects [79]. *Nod1*^*−/−*^*Nod2*^*−/−*^ mice having abundant *B. intestinihominis* demonstrate increased γδT cells in tumor beds and enhanced cyclophosphamide efficacy [79]

Local pelvic irradiation damages intestinal epithelium and barrier integrity and produce reactive oxygen species. Irradiation increase *Alistipes* and decrease *Prevotella* in mice [80]. In gynecologic cancer patients receiving pelvic radiotherapy, *Firmicutes* and *Fusobacterium* were significantly decreased [81]. In addition to reduced diversity, significant enrichment of *Clostridium IV, Roseburia*, and *Phascolarctobacterium* was associated with radiation enteropathy in pelvic cancer patients [82]. The effects of total body irradiation, which is a preparative regimen for allo-HSCT that causes dysbiosis and gastrointestinal toxicity, is discussed in more details in the allo-HSCT section below.

### Immunotherapy

**Fig. 2:**
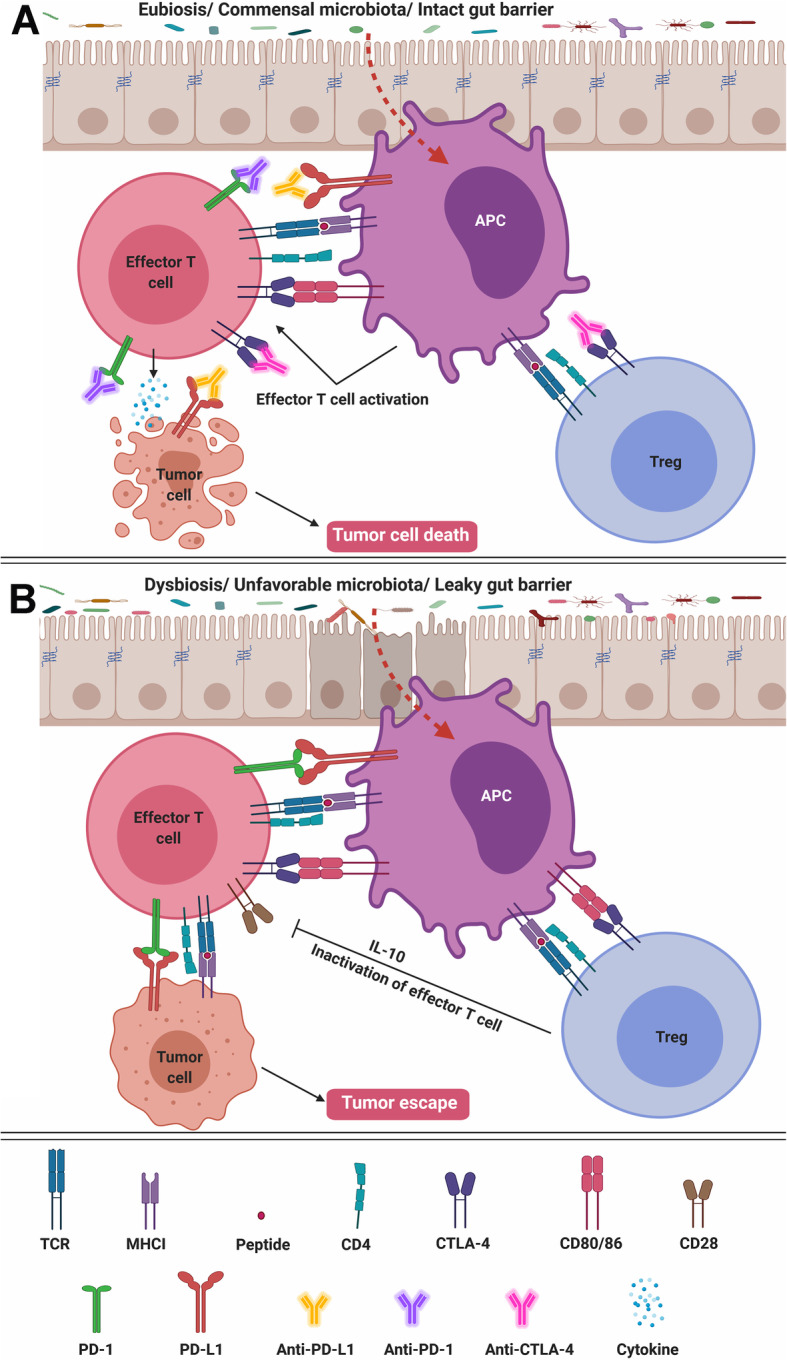
Potential antitumor immune mechanisms induced by intestinal dysbiosis. **a** In the presence of intact mucosal barrier and signals from commensal microbiota, effector T cell activation is modulated by T cell receptor (TCR) ligation with major histocompatibility complex (MHC) class I, and co-stimulation of CD80/CD86 and CD28. Binding of cytotoxic T lymphocyte-associated antigen 4 (CTLA-4) receptor to anti-CTLA-4 antibody on T_reg_ impairs its effector T-cell inhibitory function. It also downregulates CTLA-4 expression on APC. Ligation of repressive receptor programmed death receptor 1 (PD-1) and its ligand PD-L1 to anti-PD-1 and anti-PD-L1 antibodies, respectively, activate effector T-cell proliferation and function. Activated effector T cells interact with tumor cells and release cytokines to induce tumor cell death. **b** Signals from unfavorable microbes due to dysbiosis upregulates CTLA-4, PD-1 and PD-L1 expression to inhibit T-cell activation. CTLA-4 on T_reg_ binds to CD80/CD86 on antigen presenting cell (APC). CD80/CD86 on APC also dis-engages from CD28 and binds to CTLA-4 on effector T cells. PD-L1, the ligand of PD-1, is expressed on antigen presenting cell (APC) and tumor cells. PD-1 on effector T cells ligates to PD-L1 on APC and tumor cells. These activities inhibit effector T-cell activation, reduces immune checkpoint inhibitor (ICI) efficacy, and causes tumor escape

Cancer cells often create an immunosuppressive microenvironment to mediate tumor immune escape. This immune escape mechanism may be reversed by ICIs directed at cytotoxic T lymphocyte-associated antigen 4 (CTLA-4), programmed death receptor 1 (PD-1), or PD-1 ligands (PD-L1). Since intestinal microbes influence local and systemic antitumor immune reaction by modulating PRRs, PAMPs and DAMPs, intestinal dysbiosis may impact treatment outcome. Figure 2 illustrates how the potential mechanisms of the antitumor immune responses are downregulated by intestinal dysbiosis. The effects of intestinal microbiome on responses to ICIs have been discussed previously [83, 84]. Broad-spectrum antibiotics before, during, or after ICIs therapy alter intestinal microbiome and resulted in lower tumor response rate, inferior progression-free survival and reduced overall survival [85].

Responses to inhibition of CTLA-4 by ipilimumab in mouse models of MCA205 sarcoma, RET melanoma, and MC38 colon carcinoma were inferior in germ-free or in broad-spectrum antibiotic treated mice [86]. Poor responses were associated with decrease in intestinal *Bacteroides thetaiotaomicron*, *Bacteroides uniformis* and *Burkholderia cepacia*, and increase in *Clostridiales*. Such dysbiosis was also associated with mucosal damage and colitis. Oral feeding with either *Bacteroides thetaiotaomicron* or *Bacteroides fragilis* individually, or with a combination of *Bacteroides fragilis* and *Burkholderia cepacia* restored the antitumor effects of CTLA-4 blockade through augmentation of Th1 responses in tumor-draining lymph nodes and promotion of maturation of intra-tumoral dendritic cells (DCs). In addition, the combination treatment of *Bacteroides fragilis* and *Burkholderia cepacia* prevented intestinal damage and refractory colitis.

Fecal microbiota analysis of melanoma patients before and after ipilimumab treatment showed a change in the relative proportions of three dominant enterotype clusters [86]. Cluster A was dominated by *Prevotella* sp., whereas clusters B and C by different *Bacteroides* spp. Fecal microbiota transplantation (FMT) from patients into tumor-bearing, germ-free mice showed that only fecal material from cluster C resulted in colonization with *Bacteroides thetaiotaomicron* or *Bacteroides fragilis*, and enhanced ipilimumab response. In another study of ipilimumab in mice, vancomycin treatment resulted in a more severe manifestation of colitis, whereas oral administration of *Bifidobacterium* ameliorated the side effects [87]. Similarly, melanoma patients with increased abundance of *Bacteroidaceae*, *Rikenellaceae*, and *Barnesiellaceae* members responded better to CTLA-4 antibodies [88].

However, a different study in ipilimumab-treated melanoma patients found that *Bacteroides* spp. were associated with decreased response, whereas *Faecalibacterium* and other Firmicutes members improved clinical outcome [89]. Patients with higher abundance of *Faecalibacterium* and improved response to CTLA-4 antibodies showed higher incidence of enterocolitis and lower level of T_reg_ in peripheral blood. Thus, the beneficial effects of specific and isolated gut microbes may depend on the commensal association with other microbial species and may differ between humans and mice.

PD-1 blockade may also be modulated by intestinal microbiota. Melanoma patients who responded to PD-1 blockade had increased abundance of *Enterococcus faecium, Collinsella aerofaciens, Bifidobacterium adolescentis, Klebsiella pneumoniae, Veillonella parvula, Parabacteroides merdae, Lactobacillus* sp., and *Bifidobacterium longum, whereas in non-responders, the intestinal microbiome was enriched in Ruminococcus obeum* and *Roseburia intestinalis* [90]*. Another study found higher abundance of Faecalibacterium* species in responders, and enrichment with *Bacteroides thetaiotaomicron, Escherichia coli*, and *Anaerotruncus colihominis* in non-responders [91]. Clinically, non-small cell lung cancer (NSCLC) and renal cell carcinoma (RCC) patients experienced increased resistance to PD-1 blockade after antibiotic treatment [92]. These patients had shorter progression-free survival as well as overall survival. In this study, response to PD-1 blockade correlated with higher fecal abundance of *Akkermansia muciniphila*. FMT from responders to germ-free or antibiotic-treated mice improved the outcome of PD-1 blockade. Administration of *A. muciniphila* after FMT from non-responders restored response.

Similarly, intestinal microbiota may influence the outcome of chimeric antigen receptor T cell (CAR T) therapy. Patients with complete response to CD19 CAR T-therapy exhibited enrichment of *Oscillospiraceae, Ruminococcacaeae*, and *Lachnospiraceae in their intestinal microbiome, whereas patients who did not attain a complete response showed increased abundance of Peptostreptococcaceae* [93].

### Allogenic hematopoietic stem cell transplantation

Although allo-HSCT is effective in treating some hematological malignancies, the immunosuppressive agents, broad-spectrum antibiotics, and chemoradiation used with the transplant often induce intestinal dysbiosis, gut permeability and impaired systemic immune response. Higher microbiota diversity is associated with long-term survival, and lower diversity in gut microflora is associated with reduced overall survival and higher transplant-related mortality following allo-HSCT [94, 95]. Severe infections that occur due to intestinal dysbiosis, such as CDI and vancomycin-resistant enterococci (VRE) infections, are also associated with higher treatment-related mortality [96–99]. Allo-HSCT disrupts the equilibrium of bacterial composition in feces with a dominance of *Enterococcus, Streptococcus*, and *Proteobacteria* members [100, 101], and reduces beneficial bacteria such as *Faecalibacterium* and *Ruminococcus* [102]. Higher abundance of *Blautia* was found to be associated with improved overall survival [103]. Moreover, allo-HSCT patients with reduced risk of relapse had an enrichment of *Eubacterium limosum* [48].

One of the major complications of allo-HSCT is the development of graft-versus-host disease (GvHD). Occurrence of CDI during allo-HSCT increases the risk of GvHD. Besides the loss of overall microbial diversity, reduction in beneficial *Faecalibacterium*, *Blautia*, *Lactobacillus*, and *Ruminococcus*, and increased abundance of *Enterococcus* and *Clostridiales* was observed in GvHD [102, 104–106]. Patients without GvHD had increased abundance of Parabacteroides and Bacteroides in their pre-transplant feces [102]. In a preclinical study, reduced GvHD and improved overall survival was observed after the administration of the probiotics *Lactobacillus rhamnosus* GG alone or in combination with ciprofloxacin due to the preservation of gut mucosal integrity in the recipient mice [105]. Restoration of normal intestinal microbiome by FMT has been found to benefit patients with steroid-refractory GvHD [107, 108]. Multiple clinical trials are currently ongoing to investigate how manipulation of gut microbiota using dietary intervention and FMT might reduce the risk of GvHD.

## Manipulation of intestinal microbiome and barrier to improve outcome of cancer therapeutics

**Fig. 3:**
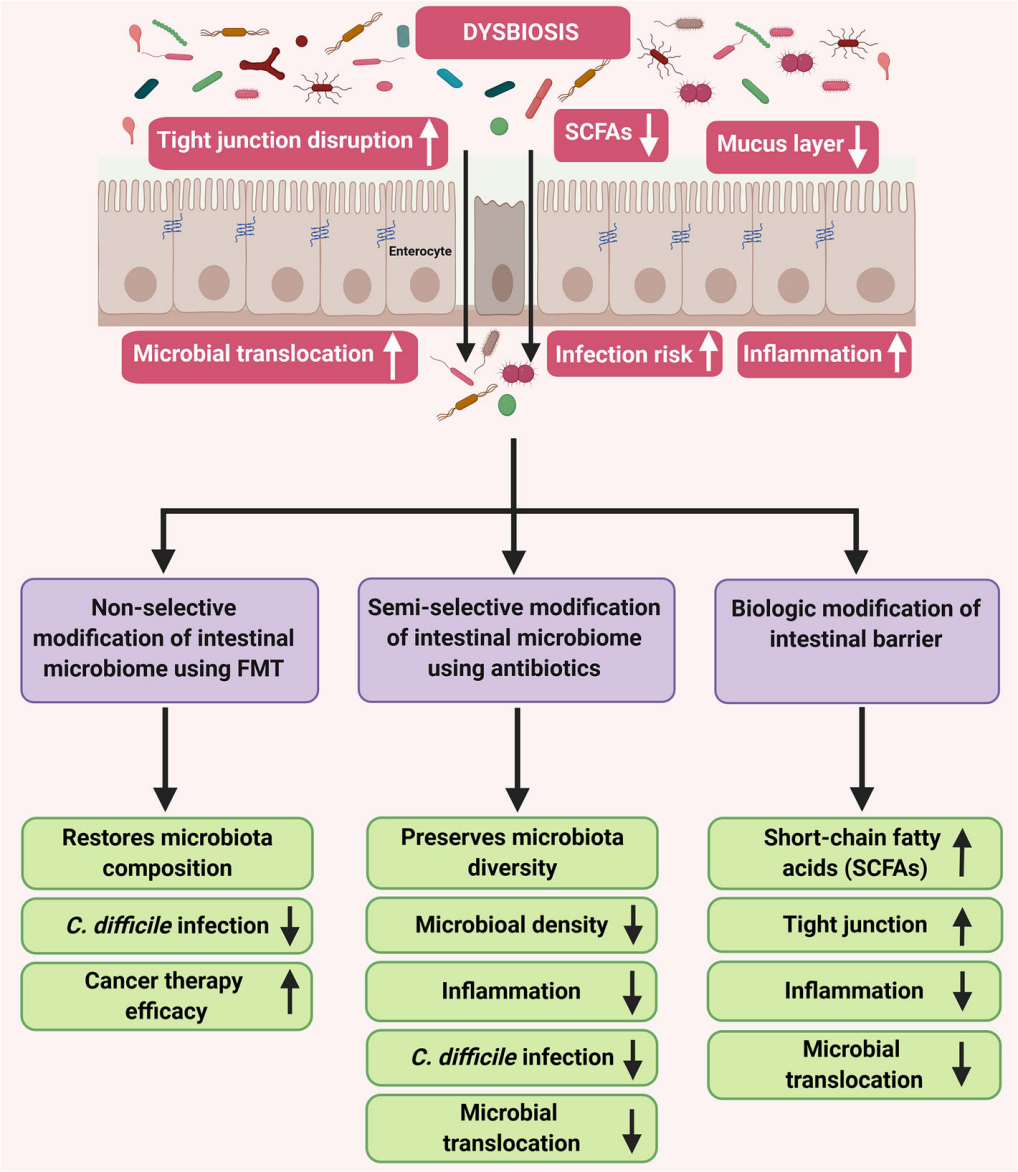
Schematic representation of three approaches to manipulate intestinal microbiome and barrier to enhance cancer therapeutic outcomes. (Short-chain fatty acids (SCFAs); Fecal microbiota transplantation (FMT))

If intestinal dysbiosis and its associated increased gut permeability are associated with cancer development, and therapy-related complications, and treatment outcomes, it follows that intervention of the intestinal microbiome and/or gut barrier may alter cancer outcome. In this section, we will explore three broad approaches (Fig. 3) that might be investigated: 1) Non-selective modification of intestinal microbiome using FMT; 2) Semi-selective modification of intestinal microbiome using antibiotics; and 3) Biologic modification of intestinal barrier. We will discuss the challenges and obstacles each of the approaches may encounter.

### Non-selective modification of intestinal microbiome using FMT

Modification of the intestinal microbiome is theoretically best accomplished by FMT. Unmanipulated FMT will not only replete the dysbiotic intestinal microbiome with the deficient microbes but also allow the re-establishment of a finely balanced microbial community. It may also re-establish/repair the intestinal barrier. FMT has been applied successfully to patients with antibiotic-resistant recurrent CDI [109–111]. Its restricted applicability due to the invasive nature of the treatment has recently been overcome with the introduction of capsule FMT. Preclinical studies have demonstrated the potential value of FMT in improving cancer outcome. *Nod2*-deficient mice that develop dysbiosis, spontaneous intestinal inflammation and increased risk of CRC, were partially protected from CRC after fecal transfer from wild-type mice [112]. When fecal material from metastatic melanoma patients undergoing anti-PD-1 therapy were transplanted into germ-free mice, feces enriched in *Faecalibacterium* species from responders elicited reduced tumor growth and improved anti-PD-1 treatment response [91]. Similar beneficial effects of FMT in immunotherapy efficacy were observed in NSCLC and RCC [92].

Steroid-refractory acute GvHD that occurred in patients who developed the complication following allo-HSCT for hematologic malignancies responded to FMT, with resolution of the GVHD in most cases [107, 108]. FMT is also effective for refractory ICI-associated colitis in cancer patients [113] and recurrent CDI in allo-HSCT recipients [97, 99, 114]. Multiple clinical trials utilizing FMT in cancer patients to improve chemotherapy or ICI efficacy in AML, metastatic melanoma, prostate cancer and RCC are underway.

Unmanipulated FMT, however, has its challenges. Serious and fatal infection may occur, especially in those with a compromised intestinal barrier. Two patients who received capsule FMT from the same donor in two independent clinical trials developed extended-spectrum beta-lactamase (ESBL)-producing *E. coli* septicemia following the procedure [115]. The patient with liver cirrhosis survived after intensive antibiotic therapy; however, the patient with myelodysplastic syndrome treated with allo-HSCT succumbed to sepsis.

It should be noted that the results of preclinical studies showing improved response to ICI following FMT involved the use of fecal material from patients who responded to ICI. Technically the FMT employed fecal materials that had already been “manipulated” in vivo, prior to donation. It, therefore, remains unknown if similar outcome would be attained with FMT from healthy donors without a cancer diagnosis and have not been previously exposed to ICI.

### Semi-selective modification of intestinal microbiome using antibiotics

Results obtained from clinical and preclinical studies showing the detrimental effects of broad-spectrum antibiotics on the outcome of ICI treatment of cancer [85] argue for the semi-selective modification of the intestinal microbiome and the restricted use of broad-spectrum antibiotics in these patients. The association between the development of CDI and VRE and the use of antibiotics also suggests the need to avoid routine use of prophylactic antibiotics in these patients. A careful choice of antibiotics may be preferable for patients who require antibiotic prophylaxis. Prophylaxis using rifaximin, a minimally absorbed broad-spectrum antibiotic, may be the answer. Rifaximin was associated with lesser disturbance to the intestinal microbial diversity, compared to ciprofloxacin and metronidazole in allo-HSCT recipients of hematologic malignancy patients [116, 117]. These patients also showed higher levels of urinary 3-indoxyl sulfate that correlated with intestinal increase in *Clostridales* involved in the production of SCFA. Transplant outcome was also more favorable, with lower incidence of GvHD and transplant-related mortality. Rifaximin prophylaxis to reduce anticancer treatment-associated gastrointestinal toxicity and diarrhea in colon adenocarcinoma (NCT04003181) and stage I-III human epidermal growth factor receptor 2 (HER2)-positive breast cancer (NCT04249622) clinical trials are currently active. A randomized placebo-control trial of rifaximin prophylaxis to reduce infection in CRC patients (NCT03563586) is also recruiting participants.

Other beneficial effects of rifaximin include protection against entero-infections, especially CDI, and restoration of mucosal barrier. Given the common occurrence of CDI in cancer patients during and after treatment, rifaximin prophylaxis may protect against recurrent/refractory CDI. In addition, rifaximin modulates bacterial metabolic function to preserve intestinal barrier, inhibit bacterial attachment, and reduce mucosal inflammation. In liver cirrhosis, rifaximin improves mucosal barrier function and reduce the translocation of enteric pathogens, endotoxin, and other products such as ammonia to prevent inflammation and the development of hepatic encephalopathy [118]. Similar benefits have been observed when sickle cell disease (SCD) patients were given rifaximin for 6 months [119]. There was a shift towards increased abundance of *Bacteroides* and *Akkermansia* in the intestine. Rifaximin improved intestinal permeability, decreased intestinal injury, and reduced the translocation of LPS [120]. These improvements in the intestinal pathophysiology reversed upon discontinuation of the rifaximin [121].

Although rifaximin preserves microbial diversity, improves gut barrier function and reduces inflammation, the beneficial effects are not seen universally across different disease types. When rifaximin was administered to human immunodeficiency virus (HIV) patients to modify the abundance of *Prevotella* and *Succinivibrio*, and depletion of *Bacteroides*, *Faecalibacterium*, and *Roseburia* [122], there were only marginal changes in the intestinal inflammation, microbial translocation, and T-cell activation [123]. Similarly, although rifaximin altered the microbial diversity with a reduction in *Ruminococcaceae* members and increase in *Bacteroides* in common variable immunodeficiency (CVID), the changes were transient, and rifaximin did not affect serum LPS and immune function [124].

### Biologic modification of intestinal barrier

modification of intestinal barrier has been practiced for many years using probiotic and prebiotic to promote the proliferation of intestinal microbes involved in SCFA production. However, due to variability in the contents of these agents, their microbial end-results cannot be consistently predicted.

Replenishing or enriching the intestinal microbiome with “good” microbes may be an alternative option. The Gram-negative mucin-degrading anaerobe, *Akkermansia muciniphila*, may fit the purpose. *A. muciniphila* fortifies the mucosal layer and plays an important role in the functional maintenance of the gut barrier [125]. *A. muciniphila* modulates immune homeostasis by activating TLR2-expressing cells [126]. It has a weak activating effect on TLR4 and little effect on TLR5, TLR9 or Nucleotide-binding oligomerization domain-containing protein (NOD)2 receptors. Several studies have shown an inverse relationship between diseased states and *A. muciniphila* abundance in the intestine [92, 125, 127–130]. *A. muciniphila* supplementation restores gut barrier function, improves inflammatory state, and protects against cancer, obesity, type 2 diabetes, hypertension, and liver diseases. In a proof-of-concept human trial of metabolic syndrome, the subjects given 3 months of oral supplementation of live or pasteurized *A. muciniphila* showed improvement of several metabolic parameters including insulin sensitivity [127]. In addition, *A. muciniphila*-treated patients had improved gut barrier functions, with significantly lower plasma LPS, white blood cell counts and inflammatory markers. The potential role of *A. muciniphila* in improving ICI therapy was demonstrated in NSCLC and RCC. Patients with increased abundance of intestinal *A. muciniphila* responded favorably to anti-PD-1 treatment [92]. Antibiotic-treated RET melanoma-bearing mice that did not respond to PD-1 blockade showed responsiveness after natural intestinal recolonization and supplementation of *A. muciniphila*. Similarly, in Lewis lung carcinoma mice model oral gavage of *A. muciniphila* increased the efficacy of PD-1 blockade. *A. muciniphila* induced DCs to secrete IL-12. As a result, increased CCR9^+^CXCR3^+^CD4^+^ T lymphocytes were recruited to the tumor microenvironment to augment the effect of anti-PD-1 antibody. Thus, *A. muciniphila* improves intestinal barrier function and cancer treatment outcomes.

Unfortunately, an overgrowth or over-abundance of *A muciniphila* may be detrimental. *A. muciniphila* degrades mucin. Its abundance may paradoxically induce damage to the mucosal barrier, promote intestinal permeability, and facilitate translocation of endotoxin resulting in negative pathological outcomes. Abundance of *A. muciniphila* was shown to promote epithelial access following mucus degradation and trigger lethal colitis by *Citrobacter rodentium* in gnotobiotic mice [131]. Additional negative effects of *A. muciniphila* enrichment was observed in a hormone receptor-positive breast cancer mice model [132]. Antibiotic-mediated reduction in gut microbiota diversity and enrichment of *A. muciniphila* promoted increased abundance of macrophages and inflammatory chemokines in the mammary gland, mammary tumor, and blood. There was increased metastatic dissemination of tumor cells into the blood, lymph nodes, and to lungs. The threshold dose needed for *A. muciniphila* to enhance cancer treatment response without inducing negative effects will, therefore, need to be established before any isolated supplementation or enrichment is attempted to improve the outcome of cancer therapy.

*A. muciniphila* abundantly expresses on its outer membrane a 32 kDa surface protein with pili-like structure called AMUC_1100 [126, 133]. AMUC_1100 may facilitate direct interaction between *A. muciniphila* and gut epithelial cells and modulate immune response via TLR2 pathway. Treatment of obese and diabetic mice with recombinant AMUC_1100 lowered plasma high-density lipoprotein (HDL), enhanced glucose tolerance, and improved gut permeability, and increased gut tight-junction markers claudin 3 and occludin [126]. In a mouse model of colitis-associated CRC, AMUC_1100 delayed tumor formation and reduced tumor number and size [134]. AMUC_1100 increased CD8^+^ T cells and TNF-α secretion in colon. In addition, there was a downregulation of proliferative markers γH2AX and Ki67, and reduction in PD-1+ T cells in AMUC_1100-treated CAC mice. Given the potential benefits and risks associated with *A. muciniphila* supplementation in cancer therapy, AMUC_1100 may be a safer alternative to using intact organisms.

Instead of recombinant AMUC_1100, *A. muciniphila*-derived extracellular vesicles (AmEVs) may be another option. In obese mice, AmEVs treatment improved gut barrier integrity by increasing expressions of occludin, zona occludens and claudin 5, and reduced inflammation due to less production of TNF-α and IL-6 [135, 136].

## Conclusions

Through animal and clinical studies, we have in the last decade gained tremendous insights into the role of gut microbiome in cancer development, progression, and treatment. Although there is a general consensus that certain bacteria such as *Faecalibacterium* and *A. muciniphila* are associated with superior immune response and cancer treatment efficacy, and *Proteobacteria* with poor treatment response, there is still a big knowledge gap in the mechanistic interactions of microbiota with host tissues under different conditions and with other microbes such as viruses, fungi, and parasites. Analysis of microbiome using 16 s rRNA sequencing or metagenomic shotgun sequencing will not capture all the species and thus miss those that are in minority. These minor microbial florae can significantly influence tissue homeostasis. Oversimplified associations of a group of microbes in certain diseased or therapeutic condition can be misleading.

Disease and therapeutic interventions not only cause dysbiosis and gut permeability, they also affect microbiota and epithelial barrier integrity in other sites. Therefore, the synergistic effects of microbiome of different organs in immune function regulation/dysregulation and therapeutic outcomes should also be considered. In addition, cancer treatment strategies based on gut microbiota results in humanized mice models or mice with transplanted human tumors may not be directly translational and can be confounding. Transplanted tumors may also not possess all its original characteristics necessary to influence microbiome changes and elicit unfavorable immune responses [137]. Thus, without a more comprehensive understanding of the interplay between microbiota in different tissues, and the knowledge of how to modulate them for specific immune response, it would remain a challenge to formulate an optimal cancer therapeutic strategy.

## Data Availability

Not applicable.

## Abbreviations

ALL: Acute lymphoblastic leukemia
Allo-HSCT: Allogeneic hematopoietic stem cell transplant
AmEV: *A. muciniphila*-derived extracellular vesicles
AML: Acute myeloid leukemia
APC: Adenomatous polyposis coli
ATP: Adenosine triphosphate
CAR T: Chimeric antigen receptor T
CCR: C-C Motif chemokine receptor
CDI: *Clostridium difficile* infection
CDT: Cytolethal distending toxin
CRC: Colorectal cancer
CTLA: T lymphocyte-associated antigen
CVID: Combined variable immune deficiency
CXCL: Chemokine (C-X-C motif) ligand
DAMP: Damage-associated molecular pattern
DC: Dendritic cell
DNA: Deoxyribonucleic acid
ESLB: Extended-spectrum beta-lactamase
FMT: Fecal microbiota transplant
GvHD: Graft-versus-host disease
HDL: High-density lipoprotein
HIV: Human immunodeficiency virus
HMGB1: High mobility group box 1
ICI: Immune checkpoint inhibitor
IL: Interleukin
LPS: Lipopolysaccharides
MALT: Mucosal-associated lymphoid tissue
MHC: Major histocompatibility complex
MUC: Mucin
MYD88: Myeloid differentiation primary response protein 88
NF-κB: Nuclear Factor kappa light chain enhancer of activated B cells
NKT: Natural killer T
NOD: Nucleotide-binding oligomerization domain-containing protein
NSCLC: Non-small cell lung cancer
PAMP: Pathogen-associated molecular pattern
PD: Programmed death receptor
PD-L: Programmed death receptor ligand
PPAR-γ: Peroxisome proliferator-activated receptor gamma
PRR: Pattern recognition receptor
RCC: Renal cell carcinoma
RNA: Ribonucleic acid
SCFA: Short-chain fatty acid
Tet2: Ten-eleven translocation-2
Th: T-helper
TIGIT: T cell immunoreceptor with Ig and ITIM domains
TLR: Toll-like receptor
TNF: Tumor necrosis factor
T_reg_: T regulatory
VRE: Vancomycin-resistant enterococci
